# Individual-level determinants of breast and cervical cancer screening and early testing in two regionally representative urban Indian populations

**DOI:** 10.1016/j.pmedr.2024.102883

**Published:** 2024-09-06

**Authors:** Bryan Min Feng Ooi, Luke Muschialli, Dimple Kondal, Gloria Andia, Ivy Ng Ho Tsun, Helen Ye Rim Huang, Kavita Singh, Aastha Aggarwal, Mohammed K. Ali, Nikhil Tandon, K.M. Venkat Narayan, Viswanathan Mohan, Preet K. Dhillon, Theresa W. Gillespie, D. Prabhakaran, Michael Goodman, Krithiga Shridhar

**Affiliations:** a School of Medicine, Imperial College, London, UK; b Department of Public Health and Primary Care, University of Cambridge, UK; c Centre for Chronic Disease Control, New Delhi, India; d London School of Hygiene and Tropical Medicine, UK; e Faculty of Medicine and Health Sciences, Royal College of Surgeons in Ireland, Dublin, Republic of Ireland; f Public Health Foundation of India, Gurugram, Haryana, India; g Hubert Department of Global Health, Emory University, Atlanta, GA, USA; h Emory Global Diabetes Research Center, Woodruff Health Sciences Center and Emory University, Atlanta, GA, USA; i Department of Epidemiology, Rollins School of Public Health, Emory University, Atlanta, GA, USA; j Department of Family and Preventive Medicine, School of Medicine, Emory University, Atlanta, GA, USA; k Department of Endocrinology, All India Institute of Medical Sciences, New Delhi, India; l Madras Diabetes Research Foundation (ICMR Center for Advanced Research on Diabetes), Chennai, Tamil Nadu, India; m Dr. Mohan’s Diabetes Specialities Centre (IDF Centre of Excellence in Diabetes Care), Gopalapuram, Chennai, Tamil Nadu, India; n Genentech Inc, South San Francisco, CA, USA; o Department of Hematology and Medical Oncology, Emory University School of Medicine, and Winship Cancer Institute, Atlanta, GA, USA; p Centre for Health Analytics, Research, and Trends, Trivedi School of Biosciences, Ashoka University, Sonipat, Haryana, India; q Heidelberg Institute of Global Health, Heidelberg University, Germany

**Keywords:** Cancer screening, India, CARRS, Psychosocial factors, Cancer stigma, Breast cancer, Cervical cancer

## Abstract

**Introduction:**

Region-specific data on individual factors associated with uptake of breast and cervical cancer screening or early testing in diverse Indian populations are limited.

**Aim:**

To assess the prevalence and individual determinants of uptake of breast and/or cervical cancer screening or testing among women aged 30–69 years in regionally representative populations of two large Indian cities: New Delhi and Chennai.

**Methods:**

We conducted an analysis of the cross-sectional data (2016–2017) nested within the Centre for Cardiometabolic Risk Reduction in South Asia cohort, established in 2010–2011 with 12,271 participants (5365 in New Delhi; 6906 in Chennai). Among 3310 women participants, we evaluated the associations of demographic, socioeconomic, lifestyle, medical, psychosocial, and reproductive factors with breast and/or cervical cancer screening or testing using multivariable logistic regression models with results expressed as adjusted odds ratios (OR) and 95% confidence intervals (CI).

**Results:**

At any point prior to 2016–2017, 193 women self-reported having undergone evaluations for breast and/or cervical cancer. The reasons for evaluation were ‘general examination’ or ‘physician’s advice’ (i.e., screening) or ‘being symptomatic’ (i.e., early testing). The overall prevalence was 5.8% for screening or testing and 2.5% for screening alone. Formal education (OR:1.88; 95% CI:1.12–3.15), high monthly household income (OR:2.27; 95% CI:1.59–3.25) and less ‘fear-of-judgement’ (OR:1.65; 95% CI:1.05–2.58) were positively associated with screening or testing uptake. When screening uptake was analysed separately, the results were generally similar.

**Conclusion:**

Our findings may have important implications for interventions at community-level (e.g., reducing ‘fear-of-judgement’, increasing awareness to screening programs and early symptoms) and health-system level (e.g., opportunistic screening).

## Highlights

• We analysed the factors for breast and cervical cancer screening and testing uptake.
• This study involved regionally representative women populations in two urban Indian cities.
• Overall prevalence of screening or early testing was 5.8% and screening alone was 2.5%.
• Perceived ‘fear of judgement’ might be a psychosocial barrier for low screening uptake.

## 1 Introduction

The World Health Organization’s Global Action Plan (2013–2020) recommends screening, early detection, and timely treatment of cervical, breast, oral and colorectal cancers as cost-effective interventions for reducing premature cancer mortality and incidence, particularly in low- and middle-income countries (LMICs) (World Health Organization. Global Action Plan for the Prevention and Control of Non-Communicable Diseases, 2013).

With its population of 1.4 billion people, India contributes the third highest number of new cancer cases and the second highest number of deaths due to cancer in the world (Ferlay, 2020). Over 178,000 breast cancers and nearly 124,000 cervical cancers were diagnosed and reported in India in 2020 with the age-standardized incidence of 25.8 and 18.0 per 100,000 women, respectively (Ferlay, 2020). In 2016, India responded to the growing cancer burden by launching the national screening and early detection program that focuses on oral, breast and cervical cancers for women and oral cancer for men (Guidelines, 2023, Rajaraman et al., 2015). *Screening* all healthy individuals aged 30–65 years to identify those with cancers or precancers (for cervical and oral cancers) before any symptoms appear and *early testing* those who have symptoms consistent with cancer, and linking to diagnosis and management are the two components of this program (Guidelines, 2023, Rajaraman et al., 2015, World Health Organization. Cancer - Screening and early detection; Guide to Early Cancer Diagnosis., 2017). This is a government-run program for all eligible men and women at no cost (Guidelines, 2023).

The recent national survey (2019–2021) revealed that less than 2% of presumably asymptomatic women aged 30–49 years, had taken up cervical and breast screening. These estimates were notably higher in several South Indian states where cervical cancer screening uptake ranged from 3.3% to 9.8% (Patel et al., 2022, National Family Health Survey. International Intitute of Population Sciences. Mumbai, India. Key Findings for NFHS-5, 2021). The low uptake of screening and early detection, as against 40–70% in high-income countries, (Young and Robb, 2021, World Health Organization. International Agency for Research on Cancer. Cancer Screening in the European Union, Report on the implementation of the Council Recommendation on cancer screening., 2017) is a major concern. It may render the program less effective and widen inequalities in cancer prevention and outcomes (Young and Robb, 2021).

Studies conducted in India have largely highlighted education and income as important determinants of cervical and breast cancer screening (Taneja et al., 2021, Pal et al., 2021). While low cancer literacy (Gupta et al., 2015) and socioeconomic status (Monica, 2020) are known to be associated with reduced cancer screening and early testing among women in India, other factors, such as lack of awareness or access to services, anxiety about undergoing the procedure, and cancer stigma, have not received sufficient attention (Patel et al., 2022, Mehrotra, n.d). There is emerging information that factors associated with uptake of cancer screening and early testing in India may vary across socio-geographic settings (Changkun et al., 2022).

To begin closing the existing knowledge gaps, we conducted a cross-sectional analysis of the data from a regionally representative urban Indian cohort study titled Centre for Cardiometabolic Risk Reduction in South Asia (CARRS) (Nair et al., 2012, Kondal et al., 2022). We examined the prevalence of breast and cervical cancer screening or early testing among eligible women in CARRS populations residing in two large Indian cities: New Delhi and Chennai, and investigated individual level factors (demographic, socioeconomic, lifestyle, medical, psychosocial, and reproductive factors) associated with the uptake of recommended screening or testing in this population.

## 2 Methods

### 2.1 Data sources

The details of the CARRS study were presented previously (Kondal et al., 2022). Briefly, CARRS is a population-based cohort, assembled in two waves (CARRS-1 in 2010–11 and CARRS-2 in 2015–16), of men and women aged 20 years and older in three major cities in South Asia—Chennai and Delhi in India and Karachi in Pakistan. We used a multistage, cluster random sampling design based on local administrative boundaries to recruit a cohort of adult men and women representative of the population in each city. Several assessments, including questionnaires on demographics and risk factors, anthropometry and biospecimens, were collected at baseline (all sites) and during periodic annual follow-ups (only in India). CARRS-1 was established with 16,287 participants (India: N=5365 in New Delhi; N=6906 in Chennai & Pakistan: N=4016 in Karachi).

The present analysis includes CARRS-1 data from New Delhi and Chennai collected during the fourth follow-up in 2016–2017. The analysis sample included 3310 women (excluding women who self-reported to be diagnosed with any cancer at baseline, n = 14) who were between the ages of 30–69 years at baseline in 2010–2011 and who responded to screening history related questions in 2016–2017. Additional data collected in 2016–2017 included demographic and socioeconomic variables, city of residence, lifestyle factors, family, medical and reproductive history, psychosocial factors such as quality-of-life, disability, depression, perceived cancer stigma and health rating.

### 2.2 Outcome variables

The primary outcome was self-reported ‘breast or cervical cancer screening or early testing’, defined as having undergone any evaluation for breast or cervical cancer at any point prior to the 2016–2017 interview for any reason (excerpts of questionnaire in Annexure-1). For breast cancer, the relevant tests included either a clinical breast examination (CBE) or a mammogram. For cervical cancer, the outcome was based on cytological assessment using pap smear or a visual inspection using acetic acid (VIA). Reasons for having undergone any evaluation included: (1) general health check-up; (2) doctor suggested to do the exam because of my age or family history of breast cancer; or (3) having discomfort, pain, or symptoms. The first two responses were used to ascertain the secondary outcome (i.e., a sub-set of the primary outcome), defined as ‘breast or cervical cancer screening’, among presumably asymptomatic individuals. Time since the individual underwent a screening or early testing (<1 year; 1–5 years; > 5 years) was also recorded.

### 2.3 Explanatory variables

Age was categorized as 30–39, 40–49, and 50–69 years and alternatively as 30–49 and 50–69 years. Monthly household income was dichotomized as < 20,000 and ≥ 20,000 Indian rupees (INR). For employment and education status, women were grouped as ‘employed’ or ‘homemaker’, and ‘no formal education’ or ‘school or college education’, respectively.

We used EuroQol 5-Dimensional 5-Level questionnaire to assess participant’s health-related quality-of-life. Participants rated their mobility, self-care, daily activities, pain or discomfort, and level of anxiety or depression, on a scale of 1–5 with 1 being ‘no issues’ and 5 being ‘severe issues or impairment’. Participants with a score of 2 or more, were deemed to have an impaired quality-of-life (van Reenen, 2019). Participants also rated their health on EuroQol visual analogue scale of 0–100, with 0 being the worst possible health and 100 being excellent health. Those scoring less than 80 were perceived to be in poor health (van Reenen, 2019). Depression score was derived from participant’s Patient Health Questionnaire-9 questionnaire (Kroenke et al., 2001). Participants were asked 9 questions on a 4-point scale ranging from ‘not at all’ (0 points) to ‘nearly every day’ (4 points). Participants with a total score of 4 or less were deemed to have no risk of depression, while scores of 5 or above indicated any risk of depression.

To determine perceived cancer stigma, participants were asked the following four questions:

• If someone in your community had cancer, would they tell the neighbours?
• Do people in the community avoid talking or eating with a person having cancer?
• Are people in your community afraid that cancer can spread from person to person?
• Do people in the community think that cancer is a curse or result of past sins?

Participants who responded ‘no’ to the first question, ‘yes’ to the remaining three questions, or ‘don’t know’ to any of the questions, were assigned a stigma score of 1. Participants who scored 0 on all questions were classified as espousing no cancer-related stigma. The questions were based on a validated tool with modifications to fit Indian context and was previously tested among selected CARRS cancer patients and caregivers (Squiers et al., 2021).

Participants were categorized (yes/no) on their ‘vigorous to moderate’ physical activity levels (i.e., activities that produced a substantial increase in breathing and/or heart rate when performed for more than 10 min at a time). The self-reported prevalence of tobacco in any form (n = 177) and alcohol use (n = 13) among these women was negligible and were not considered for further analysis. Additional data elements included self-reported medical history regarding hypertension, diabetes, hyperlipidaemia, heart disease, stroke, kidney disease, kidney stones, kidney failure, and cancer. All women were characterized as pre-menopausal or post-menopausal, while number of pregnancies was dichotomized as ‘3 or less’ versus ‘4 or more’.

### 2.4 Ethical considerations

Study protocol and study tools were approved by the Institutional Review Boards of Public Health Foundation of India, Gurugram (IRB00006658), All India Institute of Medical Sciences, New Delhi (IEC/NP-17/07.09.09), Madras Diabetic Research Foundation, Chennai, (MDRF/EC/EPI/2009/10) India, and Emory University (IRB00044159). During data collection, written informed consent was obtained from all participants. In addition, the study obtained regulatory approval from the Health Ministry Screening Committee of India.

### 2.5 Statistical analysis

We summarized overall data as means (standard deviations), medians (interquartile range), or as counts (percentages). The dependent variables were binary: never screened or early tested vs. ever screened or early tested for the primary outcome and never screened or early tested vs. ever screened for the secondary outcome. Distributions of demographic, socioeconomic, lifestyle, medical, psychosocial, and reproductive factors were compared by study site (New Delhi vs. Chennai) and by outcome status using t-test for means, Wilcoxon rank-sum test for medians and chi-square test for proportions.

The associations of individual-level factors with the primary outcome (screening or early testing) were further analysed using multivariable logistic regression analysis with results reported as adjusted odds ratios (OR) and the corresponding 95% confidence intervals (CI). Several known (age, education, geographical region, occupation, income) and less studied determinants of cancer screening or early testing (cancer stigma, reproductive factors, physical activity, quality-of-life) were selected for analysis based on available evidence (Young and Robb, 2021, Taneja et al., 2021, Pal et al., 2021, Williams-Brennan et al., 2012). For example, the screening uptake among women in India is reported to be higher in the 30–49-year age group compared to those over 50 years of age (Patel et al., 2022). For this reason age in the present analysis was categorized as 30–49 versus 50–69 years. The covariates were included incrementally in the models. Model-1 included age at baseline (<50 years; ≥50 years), city of residence (New Delhi; Chennai), monthly household income (<INR 20,000; ≥INR 20,000), formal education (yes; no) and employment status (employed; homemaker). Model-2 included the same variables as in Model-1 plus cancer stigma (yes; no), quality-of-life (impaired; not impaired) and physical activity status (inactive; active). Model-3 included the same variables as Model-2 variables plus menopause status (pre-; post-menopausal) and number of pregnancies (0–3; ≥4). The associations of individual-level factors with the secondary outcome (screening) were analysed separately in Model-4, which included all covariates as in Model-3. All data cleaning and descriptive analyses were performed using RStudio IDE release 2022.2.0 and all multivariable analyses were conducted using STATA-17 MP, StataCorp LLC, StataCorp. 2021.

## 3 Results

### 3.1 Overall characteristics of the participants **(**Table 1**)**

The study included 3310 women with an average age of 44.3 (±9.6) years. Most had formal school or college education (80.8%), were homemakers (78.4%), 42.7% had 4 or more pregnancies and 55.3% were postmenopausal. Over 45% of participants were physically inactive and reported having at least one condition (hypertension (26.8%), diabetes (25.5%), hyperlipidaemia (10.1%), heart disease (2.8%), stroke (0.6%), kidney disease (0.6%), kidney stones (4.4%), kidney failure (0.1%)). Less than one percent (n = 13) self-reported a cancer diagnosis during the follow-up period (2011–2017). Nearly a two-thirds (67.3%), espoused some cancer-related stigma and 65.7% had low perceived health rating. Almost 40% of participants experienced impaired quality-of-life and less than 10% were categorized ‘at risk for depression’.

### 3.2 Characteristics of the participants by region **(**Table 1**)**

Women residing in New Delhi compared to women in Chennai were older at baseline (mean age 45.4 ± 9.8 vs. 43.5 ± 9.4 years), were more likely to come from a household with ≥INR 20,000 monthly income (54.2% vs. 11.6%) but were less likely to be employed (14.1% vs. 27.5%) and had a higher proportion of persons with no formal education (24.5% vs. 15.0%). Compared to the Chennai cohort, the New Delhi group included higher percentages of women who were postmenopausal (61.3% vs. 50.7%), had 4 or more pregnancies (57.3% vs. 31.3%), and were physically inactive (63.3% vs. 31.1%). A greater proportion of women in New Delhi reported no perceived cancer stigma (70.1% vs. 3.5%) but reported impaired quality-of-life (51.8% vs. 30.1%).

### 3.3 Prevalence of cancer screening or early testing **(**Table 1**)**

Only 193 women self-reported having been evaluated for breast and/or cervical cancer at any point prior to 2016–2017. The self-reported reasons for any evaluation were ‘during general examination’ or ‘on physician’s advice due to age or family history’ (i.e., screening: 43.4%) or ‘on being symptomatic’ (i.e., early testing: 52.3%). The overall prevalence of screening or early testing was 5.8% (breast vs. cervix: 4.6% vs. 2.1%, p < 0.01; New Delhi vs. Chennai: 8.6% vs. 4.1%, p < 0.01). Less than 1% (n = 30) had ever undergone both breast and cervical cancer evaluations. The prevalence of asymptomatic screening was 2.5% (breast vs. cervix: 2.2% vs. 0.9%, p < 0.01; New Delhi vs. Chennai: 3.1% vs. 2.0%, p < 0.05). Over 60% of women had the evaluation of breast or cervix in the last 5 years. Among women who underwent screening or early testing, over 60% of women underwent mammography for breast cancer, 6% had both a mammogram and a CBE and the rest had only a CBE. Similarly, for cervical cancer all had at least one pap smear examination (n = 70) and a fourth had both a pap smear and a VIA test (n = 18).

### 3.4 Characteristics of the participants by outcome status **(**Table 2**)**

The previously screened or tested and never screened or tested women had similar age distributions and did not differ significantly with respect to employment status. Compared to women with no history of screening or early testing, previously screened or tested women were more likely to reside in New Delhi (61.1% vs. 42.7%), come from households with ≥INR 20,000 monthly income (54.4% vs. 28.8%) and have completed formal education (90.7% vs. 80.2%). While the distributions of various health-related factors, such as history of comorbidities, and quality-of-life, were similar in the two groups, previously screened or tested women were less likely to espouse cancer related stigma than women with no history of screening or testing (31.4% vs. 52.8%) and more likely to be premenopausal (51.3% vs. 44.1%).

Although more than 85% of women disagreed that people in their community would avoid socializing with cancer patients or thought cancer was infectious or a curse, over 60% answered that they would not share the personal information on cancer diagnosis with neighbours (Chennai: 95.8%; New Delhi:14.4%) (Table 1). With respect to specific components of cancer stigma, 38.9% women with previous history of screening or early testing and 61.5% of never screened or tested women reported that ‘someone in their community with cancer would not tell their neighbours’, indicating less pronounced ‘fear-of-judgement’ in the first group. By contrast, the other aspects of cancer stigma such as fear of cancer spreading, social isolation, or thinking that cancer is a curse, that could also have partly influenced the decision of non-disclosure of cancer diagnosis to neighbours, were not different between the two groups (Table 2).

**Table 1:** Characteristics of women aged 30–69 years at baseline (2010–2011) in an urban Indian population of New Delhi and Chennai, 2016–2017 (N=3310).

**Explanatory variables** **Number (%)**	**Total (N=3310)**	**Chennai (n = 1861)**	**New Delhi (n = 1449)**
**Age Group at baseline 2010–2011			
30–39	1186 (35.8%)	735 (39.5%)	451 (31.1%)
40–49	1159 (35.0%)	641 (34.4%)	518 (35.7%)
50–69	965 (29.2%)	485 (26.1%)	480 (33.1%)
**Mean age at baseline in years (±SD)	44.3 (9.6)	43.5 (9.4)	45.4 (9.8)
**Median age at baseline in years (IQR)	43.0 (37.0, 50.0)	42.0 (36.0, 50.0)	45.0 (38.0, 52.0)
**Total household income per month			
<20,000 rupees	2308 (69.7%)	1645 (88.4%)	663 (45.8%)
≥20,000 rupees	1002 (30.3%)	216 (11.6%)	786 (54.2%)
**Employment Status			
Currently employed	715 (21.6%)	511 (27.5%)	204 (14.1%)
Homemaker	2595 (78.4%)	1350 (72.5%)	1245 (85.9%)
**Education status			
No formal education	635 (19.2%)	280 (15.0%)	355 (24.5%)
School/College education	2675 (80.8%)	1581 (85.0%)	1094 (75.5%)
**Perceived Cancer Stigma^#^			
No Stigma	1082 (32.7%)	66 (3.5%)	1016 (70.1%)
Presence of stigma	2228 (67.3%)	1795 (96.5%)	433 (29.9%)
Cancer Stigma assessment based on individual self-reported questions^#^			
**If someone in your community had cancer, would they tell the neighbours? (no)	1991 (60.2%)	1783 (95.8%)	208 (14.4%)
**Do people in the community avoid talking or eating with a person having cancer? (yes)	379 (11.5%)	146 (7.8%)	233 (16.1%)
**Are people in your community afraid that cancer can spread from person to person? (yes)	409 (12.4%)	173 (9.3%)	236 (16.3%)
**Do people in the community think that cancer is a curse or result of past sins? (yes)	375 (11.3%)	143 (7.7%)	232 (16.0%)
Risk of Depression^##^			
No risk of depression	3011 (91.0%)	1707 (91.7%)	1304 (90.0%)
At risk of depression	299 (9.0%)	154 (8.3%)	145 (10.0%)
**Quality of Life Index^###^			
No Impairment	1999 (60.4%)	1301 (69.9%)	698 (48.2%)
Impairment in Quality of Life	1311 (39.6%)	560 (30.1%)	751 (51.8%)
Perceived Health Rating ^Δ^			
Low Perceived Health	2174 (65.7%)	1209 (65.0%)	965 (66.6%)
High Perceived Health	1134 (34.3%)	650 (34.9%)	484 (33.4%)
**Activity and Exercise			
Physically Active	1814 (54.8%)	1282 (68.9%)	532 (36.7%)
Physically Inactive	1496 (45.2%)	579 (31.1%)	917 (63.3%)
Self-reported history of comorbidities ^Δ Δ^			
Yes	1505 (45.5%)	855 (45.9%)	650 (44.9%)
No	1805 (54.5%)	1006 (54.1%)	799 (55.1%)
**Menopausal Status			
Pre-Menopausal	1474 (44.5%)	917 (49.3%)	557 (38.4%)
Post Menopause	1831 (55.3%)	943 (50.7%)	888 (61.3%)
**Number of Pregnancies			
0–3	1895 (57.3%)	1278 (68.7%)	617 (42.6%)
4 or more	1412 (42.7%)	582 (31.3%)	830 (57.3%)
**Cancer screening /early detection testing**			
**Never screened/early tested	3117 (94.2%)	1786 (96.0%)	1331 (91.9%)
**Ever screened/early tested for breast OR cervical cancer	193 (5.8%)	75 (4.0%)	118 (8.1%)
**Ever screened/early tested for breast AND cervical cancer	30 (0.9%)	09 (0.5%)	21 (1.4%)
*Ever screened/early tested for breast cancer ^Δ Δ Δ^	153 (4.6%)	73 (3.9%)	80 (5.5%)
**Ever screened/early tested for cervical cancer ^Δ Δ Δ^	70 (2.1%)	11 (0.6%)	59 (4.1%)
**Ever screened for breast or cervical cancer**	84 (2.5%)	38 (2.0%)	46 (3.1%)
**Type of breast cancer screening/ early detection testing (n = 153)^ô^ Clinical breast exam Mammogram Both	50 (32.6%) 93 (60.7%) 10 (6.5%)	33 (45.2%) 34 (46.5%) 06 (8.2%)	17 (21.2%) 59 (73.7%) 04 (5.0%)
**Type of cervical cancer screening/ early detection testing (n = 70) ^ô^** Pap smear Visual exam with acetic acid & Pap smear test	52 (74.2%) 18 (25.7%)	08 03	44 15
**Self-reported reason for screening (n = 193) ^ô ô^** Being symptomatic During general examination or on physician’s advice Missing	101 (52.3%) 84 (43.4%) 08 (4.1%)	32 (42.6%) 38 (50.6%) 05 (6.6%)	69 (58.4%) 46 (38.9%) 03 (2.5%)
**Time since last screening (n = 193)			
In the last 5 years >5years Missing	124 (64.2%) 39 (20.2%) 30 (15.5%)	62 (82.6%) 07 (9.3%) 06 (8.0%)	62 (52.5%) 32 (27.1%) 24 (20.3%)

*p < 0.05 **p < 0.01: p-values were from Chi Square test for the differences in proportions, *t*-test for the differences in means, and Wilcoxon Rank-Sum test for differences in medians (differences between New Delhi and Chennai). SD: Standard deviation; IQR: Interquartile range.

^#^ Self reported stigma of cancer was based on four questions: If someone in your community had cancer, would they tell the neighbours? Do people in the community avoid talking or eating with a person having cancer? Are people in your community afraid that cancer can spread from person to person? Do people in the community think that cancer is a curse or result of past sins? Participants who responded ‘no’ to the first question, ‘yes’ the remaining three questions, or ‘don’t know’ to any of the questions, were assigned a stigma score of 1. Participants who scored 0 on all questions were classified as espousing no cancer-related stigma.

^##^Risk of depression: a series of 9 questions where participants answered on a 4-point scale ranging from ‘not at all’ (0 points) to ‘nearly every day’ (4 points). Scores from all questions were added, and those scoring a total of 5 or more are deemed to be at risk of depression or anxiety.

^###^ Quality of Life index includes ratings based on a 1–5 Likert scale on questions related to mobility, self-care, usual activities, pain/discomfort, anxiety/depression, based on the EQ-5D-5L scale. Those who answered 2 or more for any questions are deemed to have an impaired quality of life.

^Δ^ Self-reported, subjective health rating on a scale from 1 to 100, (1–79 = low perceived health; 80–100 = high perceived health).

^Δ Δ^ Self reporting of the presence of two or more diseases, including hypertension, diabetes, hyperlipidaemia, heart disease, stroke, kidney disease, kidney stones, and kidney failure.

^Δ Δ Δ^ not exclusive.

**^ô^** only among screened/early tested participants (n = 193).

**^ô ô^** early detection testing included self-report of symptomatic testing; screening included self-report of screening during general health check-up or on the advice of the doctor due to age or family history.

Missing values less than 1% were not reported in the table.

**Table 2:** Characteristics of women aged 30–69 years at baseline by breast and/or cervical cancer screening/early detection testing in an urban Indian population of New Delhi and Chennai, 2016–2017 (N=3310).

**Explanatory variables** **Number; percentage (%); 95% confidence interval (CI)**	**Never screened/ early tested**		**Ever screened/ early tested^**	
Total Number of Participants	3117		193	
Age Group at baseline (2010–2011)	Number	% (95% CI)	Number	% (95% CI)
30–39	1116	35.8 [34.1,37.5]	70	36.3 [29.8,43.3]
40–49	1079	34.6 [33.0,36.3]	80	41.5 [34.7,48.5]
50–69	922	29.6 [28.0,31.2]	43	22.3 [17.0,28.7]
Mean age at baseline in years (±SD)	44.4 (9.6)		43.4 (8.9)	
**City of Residence				
Chennai	1786	57.3 [55.6,59.0]	75	38.9 [32.2,45.9]
Delhi	1331	42.7 [41.0,44.4]	118	61.1 [54.1,67.8]
**Total household income per month				
<20,000 rupees	2220	71.2 [69.6,72.8]	88	45.6 [38.7,52.7]
≥20,000 rupees	897	28.8 [27.2,30.4]	105	54.4 [47.3,61.3]
Employment Status				
Currently employed	678	21.8 [20.3,23.2]	37	19.2 [14.2,25.3]
Homemaker	2439	78.2 [76.8,79.7]	156	80.8 [74.7,85.8]
**Education status				
No formal education	617	19.8 [18.4,21.2]	18	9.3 [6.0,14.3]
School/College education	2500	80.2 [78.8,81.6]	175	90.7 [85.7,94.0]
**Perceived Cancer Stigma^#^				
No Stigma	980	31.4 [29.8,33.1]	102	52.8 [45.8,59.8]
Presence of stigma	2137	68.6 [66.9,70.2]	91	47.2 [40.2,54.2]
Cancer Stigma assessment based on individual self-reported questions^#^				
**If someone in your community had cancer, would they tell the neighbours? (no)	1916	61.5 [59.7,63.2]	75	38.9 [32.2,45.9]
Do people in the community avoid talking or eating with a person having cancer? (yes)	360	11.5[10.5,12.7]	19	9.8 [6.4,14.9]
Are people in your community afraid that cancer can spread from person to person? (yes)	390	12.5[11.4,13.7]	19	9.8 [6.4,14.9]
Do people in the community think that cancer is a curse or result of past sins? (yes)	356	11.4[10.4,12.6]	19	9.8 [6.4,14.9]
Risk of Depression^##^				
No risk of depression	2837	91.0 [90.0,92.0]	174	90.2 [85.1,93.6]
At risk of depression	280	9.0 [8.0,10.0]	19	9.8 [6.4,14.9]
Quality of Life Index^###^				
No Impairment	1885	60.5 [58.7,62.2]	114	59.1 [52.0,65.8]
Impairment in Quality of Life	1232	39.5 [37.8,41.3]	79	40.9 [34.2,48.0]
Perceived Health Rating ^Δ^				
Low Perceived Health	2045	65.6 [64.0,67.3]	129	66.8 [59.9,73.1]
High Perceived Health	1070	34.3 [32.7,36.0]	64	33.2 [26.9,40.1]
Activity and Exercise				
Physically Active	1716	55.1 [53.3,56.8]	98	50.8 [43.8,57.8]
Physically Inactive	1401	44.9 [43.2,46.7]	95	49.2 [42.2,56.2]
Self-reported history of comorbidities ^Δ Δ^				
Yes	1421	45.6 [43.8,47.3]	84	43.5 [36.7,50.6]
No	1696	54.4 [52.7,56.2]	109	56.5 [49.4,63.3]
*Menopausal Status				
Pre-Menopausal	1375	44.1 [42.4,45.9]	99	51.3 [44.5,58.6]
Post Menopausal	1738	55.8 [54.1,57.6]	93	48.2 [41.4,55.5]
Number of Pregnancies				
0–3	1783	57.2 [55.5,59.0]	112	58.0 [51.0,64.8]
4 or more	1331	42.7 [41.0,44.5]	81	42.0 [35.2,49.0]

*p < 0.05 **p < 0.01: p-values were from Chi Square test for the differences in proportions and *t*-test for the differences in means (differences between never screened/tested and ever screened/tested).

^either for breast and/or cervical cancer.

^#^ Self reported stigma of cancer was based on four questions: If someone in your community had cancer, would they tell the neighbours? Do people in the community avoid talking or eating with a person having cancer? Are people in your community afraid that cancer can spread from person to person? Do people in the community think that cancer is a curse or result of past sins? Participants who responded ‘no’ to the first question, ‘yes’ the remaining three questions, or ‘don’t know’ to any of the questions, were assigned a stigma score of 1. Participants who scored 0 on all questions were classified as espousing no cancer-related stigma.

^##^Risk of depression: a series of 9 questions where participants answered them on a 4-point scale ranging from ‘not at all’ (0 points) to ‘nearly every day’ (4 points). Scores from all questions were added, and those scoring a total of 5 or more are deemed to be at risk of depression or anxiety.

^###^ Quality of Life index includes ratings based on a 1–5 Likert scale on questions related to mobility, self-care, usual activities, pain/discomfort, anxiety/depression, based on the EQ-5D-5L scale. Those who answered 2 or more for any questions are deemed to have an impaired quality of life.

^Δ^ Self-reported, subjective health rating on a scale from 1 to 100, (1–79 = low perceived health; 80–100 = high perceived health).

^Δ Δ^ Self reporting of the presence of two or more diseases, including hypertension, diabetes, hyperlipidaemia, heart disease, stroke, kidney disease, kidney stones, and kidney failure.

**Table 3:** Adjusted odds ratio (OR, 95% confidence intervals, CI) for the individual-level factors for breast and/or cervical cancer screening/early detection testing among women aged 30–69 years at baseline (2010–2011) in an urban Indian population of New Delhi and Chennai, 2016–2017 (N=3310).

Explanatory Variables	Model 1 OR (95% CI) (n = 3310)	Model 2 OR (95% CI) (n = 3310)	Model 3 OR (95% CI) (n = 3303)	Model 4 OR (95% CI) (n = 3195)
Age at baseline (2010–2011)				
Age 50–69	Reference	Reference	Reference	Reference
Age 30–49	1.57 (1.10–2.25)*	1.56 (1.08–2.25)*	1.35 (0.88–2.07)	1.29 (0.70–2.38)
City of Residence				
Chennai	Reference	Reference	Reference	Reference
Delhi	1.54 (1.08–2.19)*	1.12 (0.68–1.82)	1.12 (0.68–1.85)	0.67 (0.30–1.47)
Total Household Income per month				
<20,000 rupees	Reference	Reference	Reference	Reference
≥20,000 rupees	2.27 (1.61–3.21)***	2.27 (1.59–3.24)***	2.27 (1.59–3.25)***	3.67 (2.15–6.28)***
Education Status				
No Formal Education	Reference	Reference	Reference	Reference
Underwent Formal Education	2.04 (1.23–3.40)**	1.91 (1.14–3.18)*	1.88 (1.12–3.15)*	4.57 (1.41–14.80)*
Employment Status				
Employed	Reference	Reference	Reference	Reference
Homemaker	1.05 (0.72–1.54)	1.07 (0.73–1.57)	1.07 (0.73–1.57)	0.96 (0.55–1.65)
Activity and Exercise				
Physically Inactive		Reference	Reference	Reference
Physically Active		1.20 (0.87–1.67)	1.17 (0.84–1.63)	1.39 (0.85–2.27)
Cancer Stigma^#^				
Presence of Cancer Stigma		Reference	Reference	Reference
No stigma		1.67 (1.07–2.62)*	1.65 (1.05–2.58)*	1.84 (0.89–3.80)
Quality of Life^##^				
No Impairment		Reference	Reference	Reference
Impairment in Quality of Life		1.02 (0.75–1.40)	1.05 (0.77–1.45)	0.80 (0.49–1.29)
Menopausal Status				
Pre-Menopausal			Reference	Reference
Post-Menopausal			0.78 (0.55–1.12)	1.00 (0.59–1.69)
Number of pregnancies				
0–3			Reference	Reference
≥4			1.01 (0.73–1.38)	1.13 (0.71–1.81)

Odds ratio from logistic regression models *p < 0.05, **p < 0.01, ***p < 0.001.

Models1-3: never screened or early tested versus ever screened or early tested (primary outcome).

Model 4: never screened or early tested versus ever screened (secondary outcome).

Model 1: adjusted for age at baseline (<50 years/≥50 years), city of residence (New Delhi/Chennai), monthly household income (<INR 20 K/≥INR 20 K), formal education (yes/no) and employment status (employed/homemaker).

Model 2: adjusted for Model 1 variables and cancer stigma (yes/no), quality-of-life (impaired/no impairment) and physical activity status (inactive/active).

Model 3: adjusted for Model 2 variables and menopausal status (pre-/post-menopausal) and number of pregnancies (0–3/≥4).

Model 4: adjusted for model 3 variables.

^#^ Self reported stigma of cancer was based on four questions: If someone in your community had cancer, would they tell the neighbours?

Do people in the community avoid talking or eating with a person having cancer? Are people in your community afraid that cancer can spread from person to person? Do people in the community think that cancer is a curse or result of past sins? Participants responded ‘no’ to the first question, ‘yes’ the remaining three questions, or ‘don’t know’ to any of the questions, were assigned a stigma score of 1. Participants who scored 0 on all questions were classified as espousing no cancer-related stigma.

^##^ Quality of Life index includes ratings based on a 1–5 Likert scale on questions related to mobility, self-care, usual activities, pain/discomfort, anxiety/depression, based on the EQ-5D-5L scale. Those who answered 2 or more for any questions are deemed to have an impaired quality of life.

### 3.5 Association of various risk factors with cancer screening or early testing **(**Table 3**)**

While the baseline age under 50 was significantly associated with higher screening or testing uptake in partially adjusted Model-1 and Model-2 (OR=1.57, 95% CI: 1.10–2.25; OR=1.56, 95% CI: 1.08–2.25), the association was no longer significant once reproductive factors were included in fully adjusted Model-3 (OR=1.35, 95% CI: 0.88–2.07). Similarly, the association with residence in New Delhi (vs. Chennai) was rather pronounced in Model-1 (OR=1.54, 95% CI: 1.08–2.19) but was no longer evident in Model-2 and Model-3 after adjusting for cancer stigma and other lifestyle and reproductive factors variables. On the other hand, monthly family income of at least INR 20,000 remained a strong independent determinant of screening or early testing in all three models with OR (95% CI) estimates of 2.27 (1.61, 3.21) in Model-1, 2.27 (1.59, 3.24) in Model-2 and 2.27 (1.59, 3.25) in Model-3. The corresponding results for having formal education were also consistently significant with OR ranging from 1.88 (95% CI: 1.12–3.15) in Model-3 to 2.04 (95% CI: 1.23–3.40) in Model-1. In the fully adjusted Model-3, odds of previous screening or testing were high among women demonstrating no cancer stigma (OR=1.65; 95% CI: 1.05, 2.58) relative to those who espoused at least some form of stigma. In Model-4 the independent variables were the same as in Model-3, but the outcome was restricted to screening, defined as cervical or breast cancer evaluation in presumably asymptomatic women. The overall findings in fully adjusted Model-4 (screening alone) were generally similar to Model-3 (screening or testing), but the associations with higher income and education were stronger than in Model-3. Also, for screening alone, the associations with age and residence were not evident in partially adjusted models (*data not shown*).

## 4 Discussion

In this cross-sectional study of screening-age women residing in two geographically and demographically diverse urban Indian cities, the self-reported uptake of screening or early testing for breast and cervical cancer was rather low (6%) and even lower (2.5%) when restricted to screening. Women with formal education, and those coming from households with relatively high income were about twice as likely to report previous screening or testing compared to women with no formal education and those of lower socioeconomic status. This socioeconomic disparity became even more pronounced when the outcome was defined more specifically as screening. The likelihood of screening or testing was also higher among women who espoused less cancer stigma, especially those who reported less ‘fear-of-judgement’. The associations with city of residence and younger age observed in partially adjusted models were no longer significant after accounting for additional covariates.

The low uptake of screening in our study (2.5%) was similar to the 1–2% nationwide averages reported in the 2019–2021 National Family Health Survey (NFHS-5) of India (Patel et al., 2022). When the data were examined by regions, the prevalence of screening in our study was somewhat higher compared with the NFHS results in New Delhi (0.7–1.5%), but much lower than the corresponding estimates in Chennai (6.3–8.3%).

While our findings cannot be directly compared to NFHS due to the differences in age distributions, timing, and areas of data collection, it is likely that the disagreement between the two data sources and the low uptake in Chennai compared to New Delhi in our study are attributable, at least in part, to the sociodemographic and psychosocial characteristics of participants. Perceived cancer stigma, driven by the ‘fear-of-judgement’, in Chennai was quite pronounced despite relatively high levels of education. The CARRS cohort in New Delhi was characterized by relatively high household income and low cancer stigma. Nevertheless, the findings need to be interpreted with caution due to self-reported information, although similar assessment was tested previously (Squiers et al., 2021). In contrast to the NFHS findings, we also observed that breast cancer screening uptake was higher than that for cervical cancer, and this observation may be due to the lack of availability of facilities for cervical cancer screening (Dhillon et al., 2020). The low prevalence of screening among CARRS study participants residing in Chennai is surprising as South Indian regions have consistently recorded higher screening uptake than the northern parts of the country (Patel et al., 2022, National Family Health Survey. International Intitute of Population Sciences. Mumbai, India. Key Findings for NFHS-5, 2021). It is worth noting that the cancer prevention and control systems in New Delhi may be comparable to Chennai, particularly in terms of health workforce and tertiary care hospitals (Selvaraj et al., 2022), availability of opportunistic screening models (Shridhar et al., 2015), and delivery of *Human Papilloma Virus* vaccine for cervical cancer (Mehrotra, n.d).

Our results confirmed previous observations that formal education and high household income are important determinants of screening uptake in both high and low-income countries (Nuche-Berenguer and Sakellariou, 2021, Lin, 2008, Petersen et al., 2022). Studies and national surveys in India further corroborate these findings, albeit with regional heterogeneity (Changkun et al., 2022, Patil et al., 2023). On the other hand, our data also show that formal education alone does not directly translate to high screening uptake, if other specific regional, structural and social-behavioural barriers are not addressed (Patil et al., 2023).

Although no single factor may be able to entirely explain observed cancer screening patterns (Williams-Brennan et al., 2012), our analysis shows that efforts aimed at reducing perceived ‘fear-of-judgement’ may be the most important determinant in improving breast and cervical screening rates. A recent qualitative study describing the lived experiences from Zimbabwe identified a lack of understanding of the cancer services as the common underlying theme in cancer stigma (Mandizadza and Moyo, 2021). While in our study cancer stigma appears to be largely driven by ‘fear-of-judgement’, other manifestations of stigma reported in India include fear of cancer transmission, social isolation, personal responsibility for the diagnosis, retribution for bad deeds, and the perceived inevitability of cancer-related disability or death (Gupta et al., 2015, Nyblade et al., 2017). Although more research into the effects of other social determinants of health is warranted, a previous ethnographic study in New Delhi demonstrated that social network and role-model facilitators may be used to motivate screening participation (Sekar P, Ghosh S, Dhillon P, Shridhar K. The dynamics of breast cancer screening approaches in urban India: An ethnographic study from Delhi. SSM - Qualitative Research in Health., 2022).

Approximately 3% of women in this study have undergone breast or cervical cancer early testing due to health complaints. Although this type of testing is less optimal than screening, it may still be lifesaving. Thus, awareness about the early symptoms, appropriate services and access to health care facilities may play a vital role in improving early testing (Patel et al., 2022).

The limitations of this study include the inability to analyse breast or cervical cancer screening separately or to examine possible effect modifiers or mediators due to small outcome numbers. Further, while the study populations represent two geographical regions, the generalisability of results to other parts of India is limited due its wide demographic and regional differences. These limitations notwithstanding, our study findings may have important community-level and possibly health-system level implications for increasing the uptake of cancer screening and early testing. These become particularly relevant in the current context where cost of screening and treatment, otherwise a common barrier for seeking care, (Gupta et al., 2015) is supported through the national program.

In conclusion, at the community level, gradual, stepwise public health messaging to reduce the ‘fear-of-judgement’ and increasing awareness about screening programs and early symptoms may improve the uptake. At the health system level, implementation of opportunistic screening for all eligible women presenting to health care facilities may offer another opportunity for improving uptake. Although limited, interventional studies from LMIC settings render support to our suggested recommendations, including improved symptom recognition for early help-seeking, awareness-based opportunistic screening (Nnaji et al., 2022, Gadgil et al., 2020), and m-health interventions for cervical cancer screening uptake (Zhang et al., 2020). These findings make a case for better understanding of region-specific factors that may include variable access to healthcare utilization, patient experience, health literacy, psychosocial and neighbourhood support.

## CRediT authorship contribution statement

**Bryan Min Feng Ooi:** Writing – review & editing, Writing – original draft, Investigation, Formal analysis. **Luke Muschialli:** Writing – review & editing, Writing – original draft, Investigation, Formal analysis. **Dimple Kondal:** Writing – review & editing, Project administration, Formal analysis, Data curation. **Gloria Andia:** Writing – review & editing, Writing – original draft, Investigation, Formal analysis. **Ivy Ng Ho Tsun:** Writing – review & editing, Writing – original draft. **Helen Ye Rim Huang:** Writing – review & editing. **Kavita Singh:** Writing – review & editing. **Aastha Aggarwal:** Writing – review & editing. **Mohammed K. Ali:** Writing – review & editing, Resources. **Nikhil Tandon:** Writing – review & editing, Resources. **K.M. Venkat Narayan:** Writing – review & editing, Resources. **Viswanathan Mohan:** Writing – review & editing, Resources. **Preet K. Dhillon:** Writing – review & editing. **Theresa W. Gillespie:** Writing – review & editing. **D. Prabhakaran:** Writing – review & editing, Supervision, Resources, Project administration. **Michael Goodman:** Writing – review & editing, Supervision, Project administration, Formal analysis, Conceptualization. **Krithiga Shridhar:** Writing – review & editing, Supervision, Project administration, Methodology, Formal analysis, Data curation, Conceptualization.

## Declaration of competing interest

The authors declare that they have no known competing financial interests or personal relationships that could have appeared to influence the work reported in this paper.

## Data Availability

The datasets presented in this article are not readily available because of privacy issues. Requests to access the datasets should be directed to the Indian principal investigator of the CARRS study.

## Appendix

CANCER SCREENING

7.12 Have you ever had your breasts examined by a physician, nurse or other health professional?

[Yes= 1; No= 2; Don’t know/ Not sure= 3].

If “2″ or “3” skip to Q7.17.

7.13 Have you ever had the following exams done by a physician, nurse or other health professional?

A. Breast examination

B. Mammogram (A mammogram is an X-ray image of your breast used to screen for breast cancer).

[Yes= 1; No= 2; Don’t know/ Not sure= 3].

If “2″ or “3” skip to Q7.17.

7.14 If yes for “Q7.13” when was the last time you had?

[<1 year ago= 1; 1–5 years ago= 2; >5 years ago= 3].

Breast examination Mammogram.

7.15 Do you know why the breast exam was done?

[Yes=1; No=2].

If “2″ skip to Q7.17.

7.16 If yes, why was this examination done?

[Yes=1; No=2; Not sure/Don’t know=3].

A. General health checkup.

B. Doctor suggested to do the exam because of my age or family history of breast cancer.

C. I was having discomfort, pain or symptoms.

D. Others.

If others, please specify.

7.17 Have you ever had the following exams for cervical cancer done by a physician, nurse or other health professional?

A. Pap smear (Cervix is the mouth of the uterus. Cervical cancer can be detected even before there are symptoms by several tests. In pap-smear a doctor takes a sample of cells from the cervix).

B. Visual exam with acetic acid (Cervix is the mouth of the uterus. Cervical cancer can be detected even before there are symptoms by several tests. In this test the doctor look at the mouth of the uterus, paint some vinegar on it, and see if there are any changes that look like cancer).

[Yes= 1; No= 2; Don’t know/ Not sure= 3].

If “2″ or “3” thank the participant and end the questionnaire.

7.18 If yes for “Q7.17” when was the last time you had?

[<1 year ago= 1; 1–5 years ago= 2; >5 years ago= 3].

Pap smear Visual exam with acetic acid.

7.19 Do you know why these exam done?

[Yes=1; No=2] .

If “2″ thank the participant and end the questionnaire.

7.20 If yes for Q7.19.

A. General health check-up.

B. Doctor suggested to do the exam because of my age or family history of breast Cancer.

C. I was having discomfort, pain or symptoms.

D. Others.

[Yes=1; No=2].

If yes for others, please specify.

PERCEIVED CANCER STIGMA.

Codes: Yes = 1; No = 2; Don’t know = 3; Refused = 4.

3.43 If someone in your community had cancer, would they tell the neighbours?

3.44 Do people in the community avoid talking or eating with a person having cancer?

3.45 Are people in your community afraid that cancer can spread from person to person?

3.46 Do people in the community think that cancer is a curse or result of past sins?
